# Effect of high-fluoride dentifrice on root dentine de-remineralization exposed to erosion challenge *in vitro*

**DOI:** 10.4317/jced.59091

**Published:** 2022-07-01

**Authors:** José Leal, Robson Ferreira, Guilherme Santana, Paulo Silva-Fialho, Leonardo Oliveira-Lima, Gláuber Vale

**Affiliations:** 1 Restorative Dentistry Department, Federal University of Piauí, Teresina, Brazil

## Abstract

**Background:**

High-fluoride dentifrice can be used to manage dental erosion; however, little is known about it effect on root dentine previously demineralized. This study evaluated the effect of high-fluoride treatment on dentin de/remineralization exposed to an erosion challenge in vitro.

**Material and Methods:**

Sound and demineralized dentine blocks were submitted to a 5-days-erosive challenge in soft drink (4/day during 90 s) and treated with fluoride solutions (0, 1,100, or 5,000 µg F/mL). After this, the percentage of surface hardness loss (%SHL) or recovery (%SHR) was calculated. Data were analyzed by one-way ANOVA and Tukey post hoc test with p fixed at 5%.

**Results:**

High-fluoride treatment was able to reduce dentine remineralization and increase mineral recovery of previously demineralized dentine compared to other treatments tested (*p*<0.05).

**Conclusions:**

High-fluoride treatment was able to increase the remineralization and reduce the demineralization of root dentine submitted to an erosive challenge in vitro, being an option for the erosion prevention/treatment.

** Key words:**Fluorides, root caries, tooth erosion, toothpastes.

## Introduction

Dental erosion may be a worldwide problem with a growing prevalence (1). Dentine substrate is deeply affected by the ingestion of acidic beverages, especially for adults and for the elderly population, which has gingival recession more commonly and consequently the exposure of cement and root dentine (2). The implication of erosion in oral health includes dentine hypersensitivity and aesthetic damage. The treatment of dental erosion is often performed through restorative techniques, even though it can be challenging and expensive (3).

Several methods can be used to prevent or delay the progression of dental erosion, such as dietary intervention, fluoride (F) use, and change in acid consumption of acid beverages and oral hygiene (4). Oral care products containing fluoride, such F-dentifrices have been recommended (5,6). However, the prevention of erosion by topical F-dentifrices seems to be only partially effective and requires an intensive fluoridation regime to achieve significant protection (7), especially for dentine substrate, which is more soluble, and therefore the erosive wear is reduced only by high fluoride products, including high F-dentifrices (8,9).

Although some studies have proved the effect of high F products on prevention of dentine erosion, there are no studies in the literature supporting the use of high fluoride dentifrice in the development of erosive lesions previously decayed. Thus, the aim of this study was to evaluate the effect of 5,000 µg F/g solution on dentine de and remineralization exposed to an erosion model.

## Material and Methods

-Dentine blocks preparation and selection

**Figure 1:**
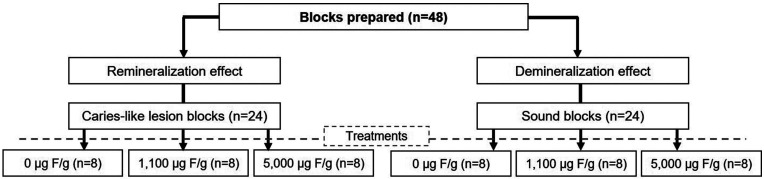
Scheme of root dentine blocks distribution among the experimental approaches and treatments.

Dentine blocks were obtained from the root of sound bovine incisors, which had already been previously disinfected in 10% formaldehyde solution, pH 7.0, for 10 days (10). Blocks were sectioned, flattened, and polished, showing final dimensions of approximately 4x4x2 mm. Baseline blocks were selected by surface hardness average (43.62 ± 4.19) employing a microhardness tester with a Knoop penetrator. Subsequently, caries-like lesions were induced (11) in half of the blocks, and then carious-like blocks were selected by percentage of surface hardness loss (%SHL) average (63.54 ± 5.92). The randomization of the blocks (n = 8) among the treatments is shown in Fig. 1. The sample size was determined based on previous findings using an equivalent experimental protocol, with a statistical power above 0.8 (10).

-Erosive challenge

The blocks were incubated in artificial saliva (0.33 g KH2PO4, 0.34 g Na2HPO4, 1.27 g KCl, 0.16 g NaSCN, 0.58 g NaCl, 0.17 g CaCl2, 16 g of NH4Cl, 0.2 g of urea, 0.03 g of glucose and 0.002 g of ascorbic acid) (5 mL/specimen) for 24 h before erosion challenges. All blocks were then subjected to five-day erosion challenge. Erosion was carried out with freshly opened bottles of Coca-Cola (Coca-Cola, pH 2.6, Brazil, 30 mL/sample, without stirring, at 25° C) four times a day for 90 seconds each. After each erosive challenge, the blocks were rinsed with distilled water (5 seconds) and transferred to artificial saliva (pH 6.8, 30 mL/sample, without stirring, at 25° C) for 1 h. After the first and last erosive challenge of each day, the blocks were treated with the treatment solution for one minute. A new aliquot of soda was used for each erosive challenge and artificial saliva was replaced daily. After the last erosive challenge of each day, the blocks were stored in artificial saliva overnight (10).

-Treatments application

The treatment solutions were prepared using artificial saliva previously described and sodium fluoride (NaF, Sigma-Aldrich, Saint Louis, MO, USA). The F concentrations of the solutions were based on the use of conventional F dentifrice (1,100 µg F/g) and high fluoride dentifrice (5,000 µg F/g) after dissolution by saliva, resulting in a final concentration of 350 µg F/g (or 0.08% NaF) and 1650 µg F/g (or 0.4% NaF) for conventional and high fluoride dentifrices, respectively. A placebo solution (without F) was used as negative control.

-Outcomes 

After the erosive challenge, the surface hardness of the blocks was measured again and the percentage of surface hardness loss of sound blocks (%SHL) was calculated by the following equation: (pre-cycling surface hardness - post-cycling surface hardness) / (pre-cycling surface hardness) x 100. The percentage of surface hardness recovery (%SHR) of carious-like blocks was calculated according to this following equation: %SHR = (post-cycling surface hardness - carious-like surface hardness) / (pre-cycling surface hardness – carious-like surface hardness) x100.

-Statistical Analysis

All data presented normal distribution of errors and were analyzed by analysis of variance (one-way ANOVA) followed by Tukey post hoc test with α fixed at 5%.

## Results

Table 1 shows the mineral changes in dentine. After allocation of the blocks among the treatment groups, neither sound nor carious-like blocks showed statistical differences in the baseline values among treatments (*p* > 0.05). After the erosive protocol, all groups differed among each other (*p* < 0.05), but the high-fluoride treatment showed less demineralization than other groups, irrespective of demineralization or remineralization approach, which reflected in the %SHL and %SHR results as well.

**Table 1:**
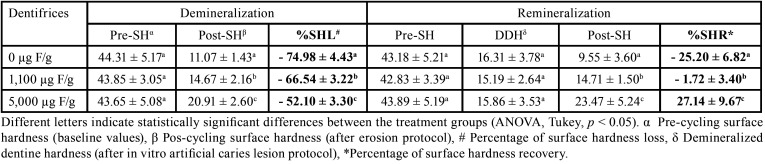
Mineral changes in dentine (Mean ± SD) according to the study approaches (demineralization or remineralization) and dentifrices after erosive protocol (n=8).

**Figure 2:**
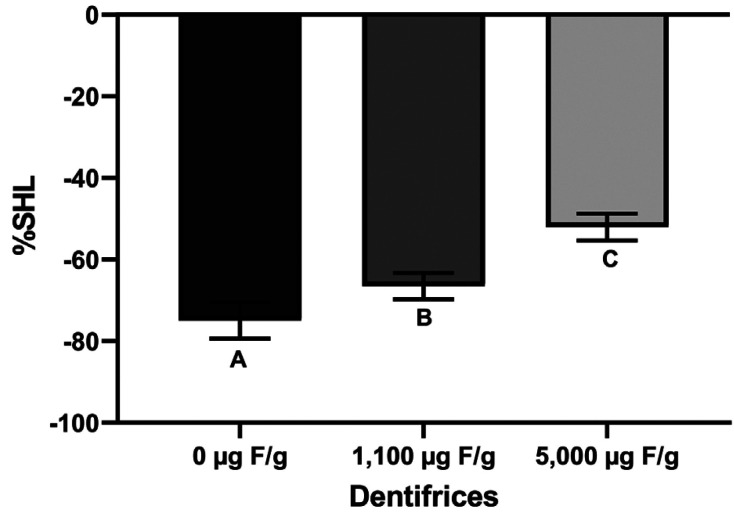
Mean (±SD) of %SHL in relation to dentifrices treatment (n=8). Different letters represent significant statistical difference (*p* < 0.05).

**Figure 3:**
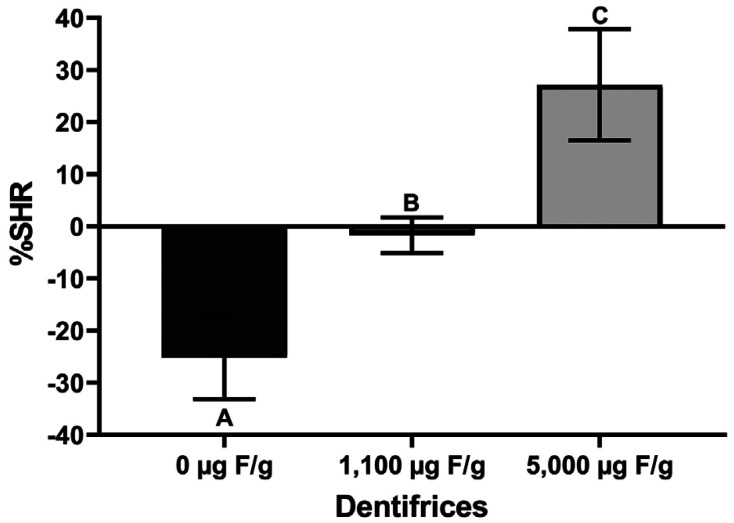
Mean (±SD) of %SHR in relation to dentifrices treatment (n=8). Different letters represent significant statistical difference (*p* < 0.05).

The effects of the treatments on the loss and gain of dentine surface hardness are shown graphically in Figs. 2 and 3, respectively. High fluoride treatment results in a lower %SHL than the conventional and placebo ones (Fig. 2, *p* < 0.05). Likewise, the 5,000 ug F/g treatment was able to increase the mineral recovery (%SHR) of the previously demineralized dentine compared to the 1,100 ug F/g treatment, which appears to maintain the mineral loss. On the other hand, placebo treatment increased mineral loss (Fig. 3). All groups differed statistically (*p* < 0.05).

## Discussion

In the present study, erosive demineralization was produced by a cola drink, one of the most consumed soft drinks that exhibits erosive potential (12). Erosive challenge was performed for 90s, 4 times per day, which is representative of the fast consumption of an acidic beverage (13). Between erosive challenges, specimens were remineralized in artificial saliva to simulate the pH changes that occur in the oral environment. Also, the use of F-treatments was evaluated and showed an overall positive effect on dentine erosion reduction. The protocol used to produce artificial lesions was succeed since the dentine blocks had a hardness loss of approximately 64% (Table 1).

The results of our study showed that high-fluoride treatment reduced the demineralization of sound dentine exposed to erosive challenge (Table 1, Fig. 2). These results are in line with others *in vitro* and in situ studies, which showed a greater effect of 5,000 µg F/g dentifrice on preventing dentine demineralization compared to the 1,100 µg F/g dentifrice (8,14). It could be explained by the formation of a Calcium Fluoride (CaF2)-like layer which behave as a physical barrier, providing some additional mineral to be dissolved during an acid challenge before the underlying dentine is attacked (15).

Although the mechanism of action of fluoride on enamel and dentin are quite similar, the concentration of CaF2-like deposits in dentin was found to be sevenfold higher than it was in enamel, which could be explained by the smaller size of the hydroxyapatite crystals in dentin, resulting in a larger surface area, therefore a more reactive mineral phase (16). Apart from that, the formation of the CaF2-like layer and its protective effect on demineralization also depends on the pH and F concentration of the agent (17), which corroborates the results found as the higher F concentration treatment led to a less demineralization.

However, the most expressive result was the mineral gain of previously demineralized dentine with the high-fluoride treatment (Table 1, Fig. 3). To the best of our knowledge, this is the first study that addresses this issue. While for caries, it has been shown that the use of high fluoride dentifrice is able to reverse primary roots caries lesions compared to a conventional dentifrice within 6 months (18); for erosion, this effect was not known. In this case, CaF2-like may act as a fluoride reservoir and during the acid challenge induces acid buffering by increasing its dissolution rate. This would increase fluoride adsorption over the crystal surfaces or accelerates surface precipitation of more acid-resistant F-containing apatites and therefore the overall stability of the hard tissue will tend to increase (17,19).

Indeed, dentin is a more acid-soluble substrate than enamel (20), resulting in more calcium being released by the low pH treatment to produce artificial lesion, which would react with fluoride and precipitates as CaF2-like material (21). However, despite these characteristics, the presence of organic matrix was found to be a key factor for the effectiveness of fluoride against dentin erosion. Organic matrix is not only capable of slowing down demineralization, but in the presence of high amounts of fluoride, it can stop the process of erosion (22). This may explain why fluoride only showed a higher protective effect for the artificial caries specimens, which probably had the organic matrix exposed because of the initial exposure to acid to create an initial lesion.

This study has limitations inherent to *in vitro* experiments, since the erosion protocol occurred under controlled conditions in the laboratory, without the complexity present in oral cavity. Also, the abrasion provoked by the toothbrushing was not evaluated in this static model. However, the model used was responsive to the treatments applied, and the outcome used (surface hardness) has been used as indicator of mineral loss and gain in erosion studies (12,13).

## Conclusions

Within the limitations of an *in vitro* study, the treatment containing 5,000 µg F/g was able to reduce dentine demineralization and increase the remineralization of previously demineralized dentine undergoing an erosive challenge. Its use can be adopted in the prevention/treatment of these lesions, but it needs in situ and *in vivo* further studies.
